# Enhanced Recovery after arthroplasty surgery

**DOI:** 10.1080/17453674.2020.1763565

**Published:** 2020-05-14

**Authors:** Rob G H H Nelissen

**Affiliations:** Leiden University Medical Center, Albinusdreef 2, 2333 ZA Leiden, the Netherlands

Terminology for accelerated postoperative functional recovery of patients after arthroplasty surgery has changed from fast track surgery, rapid recovery surgery, enhanced recovery after surgery (ERAS), as well as initiatives like getting it right first time (GRIFT). All aim to have the most favorable treatment course (i.e. optimal benefit/risk balance) for the patient after surgery, in the shortest length of hospital stay (LOS). But enhanced recovery is more than just reducing length of hospital stay, admitting patients the day of surgery, mobilize them within 3-6 hours after surgery and get patients in control of daily living activities. The aim of this perioperative and rehabilitation enhanced recovery period is to “cure” a patient from its pathology or symptoms including the stress invoked by the treatment as such. For that matter, great diversity exists after arthroplasty surgery, some patients perform excellent, and are referred to as examples of “best practice” outcome, while others are worse after surgery not expected by the patient or clinician.

The initiative of enhanced recovery after surgery is based on (patho)physiological principles on how a human being responds to surgical stress. Henrik Kehlet, a Danish surgeon, has addressed this complex pathophysiological phenomenon in the perioperative period extensively since the late 80’s up to present day in collaboration with all surgical disciplines (Kehlet 1997). A such he used existing basic knowledge to change thinking in clinical practice. Evidence-based medicine is the conscientious, explicit and ethical use of best available evidence to have optimal care of individual patients. For that matter knowledge on pathophysiological principles, recovery after stress, the impact of stress on the patient was nothing knew, but the combination throughout the overall perioperative period was rather new.

The surgical stress response originates at the surgical site with an inflammatory response (IL-6, PGE), katabolic/anabolic hormonal release/body heat loss which cause more stress hormone release within the feedback loop, which causes more stress etc. The basic idea is to reduce these effects of surgical stimuli by optimizing the individual patient before, during and after surgery. Perioperative blood management is only one important factor, which was evaluated in the largest RCT on blood management in over 2,500 patients (So-Osman et al. 2014). The integral approach on this and other perioperative factors on the patient recovery are addressed in the multimodal approach on pathophysiological responses after surgery in patients. A such enhanced recovery after surgery (ERAS), as terminology perfectly explains this complex process, is then the repair of one part of the musculoskeletal organ (e.g. hip, knee, spine etc.) of a patient. It needs a multidisciplinary collaboration between surgeon, anaesthesiologists, nursing staff, rehabilitation and the patient.

Henrik Husted, Gitte Holm, and Steffen Jacobsen evaluated predictors for length of stay and patient satisfaction after an accelerated hip and knee arthroplasty regimen (Husted et al. 2008). Their conclusion on the predictors for good outcome was “*Rather it is the sum of information given before and during the admission – regarding the intended regimen, the intended short LOS, and the motivation of the patient to actively participate – that results in shorter LOS compared to conventional surgical tracks*”.

They combined several modalities of knowledge at that time (2003–2006), including extensive education to the patient and their family, in 712 unselected patients. The active participation of the patient is his or hers recovery was sensed to be important. During the last decade the patient has been transformed from a passive human being undergoing a treatment into a more active stakeholder, engaging into his or hers recovery. Engagement in possible treatment options with the consulting clinician, also stimulated the development of patient reported outcome (PRO) measures as well its value as outcome measures.

Since Husted’s paper (Husted et al. 2008), a multitude of articles on enhanced recovery have been written. Recently data from over 400,000 hip and 400,000 knee arthroplasty patients from the National Joint Registry (NJR) of the UK & Wales showed that across the country during 10 years a trend towards shorter LOS, and a decrease of 90 days complications to 1.6% irrespective of the implementation of ERAS (Judge et al. 2020). Notably, quality of life scores did not show clinical important differences between ERAS and non-ERAS hospitals. Comparable results were found with data from the Swedish hip (SHAR) and knee (SKAR) registry (Berg et al. 2020). Nevertheless it seems obvious that the focus of the last 2 decades on enhanced recovery in a multimodal approach is very likely to be instrumental to all (also non-ERAS) hospital protocols. Standard clinical practice as such does not exist, it is an adaptive process but evidence on the enhanced recovery has been evaluated recently (Wainwright et al. 2020).

In 2020 enhanced recovery after surgery protocols can be considered as least as good as conventional care for patients, since conventional care as such is not similar to conventional care of 2000, but is today comparable to or almost similar to enhanced recovery. Henrik Kehlet integrated his ideas and research, “logos”, on the pathophysiological response of the human body to a surgical intervention, by his and other authors “pathos” the topic of enhanced recovery after surgery and thus rapid functional recovery became viral. Essentially, the aim is “ethos” recovery of our patients.

Rob G H H Nelissen *Leiden University Medical Center, Albinusdreef 2,* *2333 ZA Leiden, the Netherlands* *E-mail:*r.g.h.h.nelissen@lumc.nl
